# Capnocytophaga canimorsus endocarditis following a dog lick – a case report

**DOI:** 10.1099/acmi.0.001113.v3

**Published:** 2026-01-28

**Authors:** Winifred Garr, Marta Verga, James O'Neill, Jonathan Sandoe, Kalyana Javangula

**Affiliations:** 1 Department of Microbiology, Leeds Teaching Hospitals NHS Trust, Leeds, UK; 2 Department of Cardiology, Leeds Teaching Hospitals NHS Trust, Leeds, UK; 3 Department of Microbiology, Leeds Teaching Hospitals NHS Trust, University of Leeds, Leeds, UK; 4 Department of Cardiothoracic Surgery, Leeds Teaching Hospitals NHS Trust, Leeds, UK

**Keywords:** bacteraemia, *Capnocytophaga canimorsus*, dog lick, infective endocarditis

## Abstract

*Capnocytophaga canimorsus* is a fastidious Gram-negative bacterium found in the mouths of dogs and cats. It is a rare cause of infective endocarditis, when it is often associated with dog bites. We present a case of *C. canimorsus* infective endocarditis complicated by aortic regurgitation and root abscess in a patient with a history of previous infective endocarditis. The patient underwent redo aortic valve surgery with aortic valve replacement. Blood cultures and 16S ribosomal ribonucleic acid gene amplification and sequencing from the excised valve tissue confirmed *C. canimorsus* as the cause. The patient was treated with beta-lactam antibiotics and discharged home. Rather than secondary to a dog bite, infection most likely occurred due to a dog licking an open wound. It is important to remember that dog contact, often perceived as innocuous, such as being licked, can be a source of serious infection, particularly in the context of an open wound. Over a third of households in the UK own a dog as a pet. With *C. canimorsus* infections thought to be on the rise, in part due to increased pet ownership, there is a need to ensure pet owners, particularly those at risk of infections and chronic skin wounds, are educated on such risks and the appropriate preventative steps.

## Data Summary

No data were generated or reused in the research.

## Introduction

*Capnocytophaga canimorsus* is a fastidious Gram-negative bacterium [1] which is part of the normal oral flora of dogs and cats. It can sometimes cause human infection, particularly in the immunocompromised [1,2], and is a rare cause of infective endocarditis (IE), when it is often associated with dog bites [2,3]. We describe the case of a patient with no known immunocompromise presenting with *C. canimorsus* prosthetic aortic valve IE complicated by moderate aortic regurgitation and root abscess.

## Case report

A 55-year-old woman attended the emergency department, brought in by ambulance with fever, confusion, tachycardia and breathlessness. She had been experiencing night sweats for several weeks, associated with anorexia, chest pain, lethargy and weight loss. She reported feeling ‘not herself’ for months, suffering panic attacks and tremors described as cold, shaking episodes.

She had a significant past medical history of rheumatic fever in childhood and a previous episode of culture-negative native aortic valve IE with a suspected aortic root abscess, requiring surgical repair of the aortic root and valve (via patch) 39 years prior to presentation. This had been complicated by an embolic stroke, with residual limb weakness and post-stroke epilepsy. She lived alone with her dog.

Clinical assessment identified an ejection systolic murmur over the aortic area, no early diastolic murmur and new atrial fibrillation. Clinical biochemistry revealed anaemia (Hb 89 g l^−1^, reference range: 115–160 g l^−1^), hypo-albuminaemia (24 g l^−1^, reference range: 35–50 g l^−1^) and raised inflammatory markers: C-reactive protein (CRP) 126 (reference range: <10 mg l^−1^) and white blood cell count 15.15×10^9^/l (reference range: 4–11×10^9^/l) (Table 1). She was treated as having probable prosthetic aortic valve IE and initially received empirical flucloxacillin and gentamicin.

**Table 1.** Trend in laboratory blood results over the course of the hospital admission

Day of hospital admission	Laboratory blood result (reference range)			
	Haemoglobin *(*115–160 g l^−1^)	White blood cell count (4–11×10^9^/l)	CRP (<10 mg l^−1^)	Albumin (35–50 g l^−1^)
0	89	15.15	126	24
4	86	11.26	126	24
7	No result available	No result available	128	20
10	83	7.68	141	No result available
22 (1 day post-surgery)	91	6.75	46	26
28 (1 week post-surgery)	109	6.41	No result available	27

**Fig. 1.**
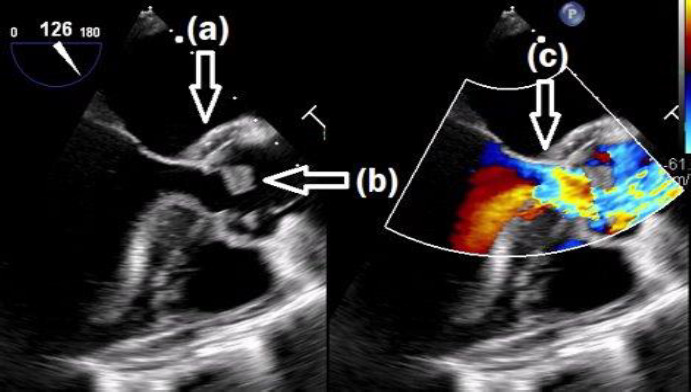
Echocardiogram image showing (**a**) root abscess, (**b**) vegetation and (**c**) aortic regurgitation.

Trans-oesophageal echocardiography identified severe aortic regurgitation, a large vegetation on the non-coronary cusp of the aortic valve and a thickened aortic root with an echo-free space, suggestive of an aortic root abscess (Fig. 1). This confirmed the diagnosis of prosthetic valve IE (PVIE).

Peripheral blood cultures (three sets) taken on the day of admission flagged positive after 3 days with growth in both the aerobic and anaerobic bottles. The morphology on the Gram stain was described as ‘possible clusters of fine Gram-negative bacilli’. There was a persistent fever, accompanied by static CRP and albumin levels (Table 1). The antibiotics were changed to treat for possible HACEK-related PVIE. Flucloxacillin was stopped and switched instead to cefotaxime, and the gentamicin was continued for its synergistic effect. On day 7 of the hospital admission (reflecting its fastidious nature), a mass spectroscopy (MALDI-TOF-MS) result from growth on the initial blood culture identified *C. canimorsus*. The cefotaxime was changed to benzylpenicillin, and the gentamicin was stopped – an empirical choice based on the identification of the organism, whilst susceptibility results were awaited. Susceptibility testing identified the *C. canimorsus* as sensitive to penicillin, sensitive to cefotaxime and resistant to gentamicin. Repeat blood cultures taken on day 3, flagged positive after 48 h with growth additionally later identified as *C. canimorsus*. Further repeat blood cultures taken on days 4, 8 and 16 of admission demonstrated no growth (5 days incubation for the blood culture taken on day 4; and prolonged incubation (10 days) for the blood cultures taken on days 8 and 16). Each of the blood culture sets was taken following an episode of pyrexia in the patient.

By day 12, there were ongoing symptoms of fever, anorexia, lethargy and breathlessness. The CRP had risen to 141 mg l^−1^ and the albumin had fallen to 20 g l^−1^, suggestive of ongoing uncontrolled infection. The benzylpenicillin was changed back to cefotaxime, and a decision was made for source control through surgical intervention.

On re-visiting the history, it transpired that the patient’s dog had a habit of licking open wounds. There had been a fall at home in the weeks prior to admission, resulting in a superficial pre-tibial laceration, and the dog had licked it.

The patient underwent redo cardiac surgery with a tissue aortic valve replacement. Aortotomy identified vegetations on the non- and left coronary cusps (the cusps with previous patch repair) and a normal-looking aortic root with no evidence of abscess. There were no organisms seen on the Gram stain performed on the tissue from the aortic valve and no growth from its culture. 16S ribosomal ribonucleic acid sequencing (16S PCR) from the excised valve tissue performed locally confirmed *C. canimorsus* with 99.9% similarity. There was a marked clinical improvement post-operatively. The patient’s appetite and energy levels returned to baseline. There was no relapse of febrile illness, and the CRP had decreased to 46 mg l^−1^. She completed a 28-day total antibiotic course and was discharged home.

## Discussion

*C. canimorsus* is a rare cause of IE, prior literature comprising a few case reports [2,4]. Though case numbers are small, *C. canimorsus* IE typically presents with fever and with sepsis in approximately a third of cases [2]. This is consistent with our case, and it is noteworthy that the patient’s reported ‘panic attacks’ and ‘tremors’ were more likely to be rigors. Invasive infection caused by *C. canimorsus* is seen more often in settings of immunosuppression and asplenia (anatomical or functional, e.g. in the context of alcohol excess). This was not the case in our patient, which was unusual.

*C. canimorsus* IE appears to affect the aortic valve most commonly [2] and is typically associated with dog bites [5]. However, as with our case, it is important to remember that dog contact perceived as seemingly innocuous, such as being licked, can be a significant source of infection, particularly in the context of an open wound acting as a portal of entry. Over a third of households in the UK own a dog as a pet [6,7]. With *C. canimorsus* infections thought to be on the rise in part due to increased pet ownership [2], there is a considerable safety benefit to be had in ensuring pet owners are educated on such risks and the appropriate preventative steps.

The Gram stain appearance of the organism grown from the initial blood culture was unusual and unfamiliar, in keeping with *C. canimorsu*s being an organism uncommonly encountered. Consistent with its recognized fastidious nature [4], it was not an easy organism to grow, posing challenges in its recognition for the laboratory. The appearance on the initial Gram stain was from the aerobic bottle (having flagged positive nearly 12 h before the anaerobic bottle) and it was difficult to interpret. Multiple agar plates were set up to cater for a broad range of potential organisms. No organisms were seen on the Gram stain from the anaerobic bottle the following day, and there was no growth from the aerobic bottle either. Both bottles and the agar plates were re-incubated, with a plan for terminal subculture at 10 days (it was at day 7 that there was a positive MALDI-TOF-MS result). This highlights just how easily *C. canimorsus* can be potentially missed or misidentified, and the utility and importance of prolonged incubation of cultures for such organisms.

There were several additional peripheral blood cultures taken during the admission that demonstrated no growth even with prolonged culture, despite recurrent pyrexia in the patient. This may have been the result of a combination of antibiotic exposure throughout the admission and the fastidious nature of *C. canimorsus* [4].

Antimicrobial susceptibility was inferred using reference data including pharmacokinetic and pharmacodynamic cut-off values in guidance provided by the European Committee on Antimicrobial Susceptibility Testing in cases where clinical breakpoints are not available [8]. MALDI-TOF-MS of the bacterial culture, and 16S PCR from the excised valve tissue played a key role in the identification and confirmation of the diagnosis, consistent with that documented in the literature [2]. The ability for the 16S PCR testing to be performed in-house facilitated more timely confirmation of the diagnosis of this uncommon fastidious organism. *C. canimorsus* IE is commonly treated with aminoglycoside and beta-lactam antibiotics [2]; and the management of our patient was consistent with this, although it was interesting and unusual to note the resistance to gentamicin on antimicrobial susceptibility testing.

## Conclusion

This case highlights *C. canimorsus* as a rare but important cause of PVIE [4]. Importantly, it emphasizes that *C. canimorsus* IE can occur from canine contact other than bites (licking in this case) – contact that may be perceived as harmless by pet owners. It is important that clinicians enquire about contact with animals, including pets, during history-taking . The case also reinforces the importance of ensuring pet owners are aware of the risks and the need to avoid animal contact with open wounds.

## Abbreviations

CRP: C-reactive protein
IE: infective endocarditis
MALDI-TOF-MS: MALDI-TOF mass spectroscopy
PVIE: prosthetic valve infective endocarditis
